# A new approach of using organ-on-a-chip and fluid–structure interaction modeling to investigate biomechanical characteristics in tissue-engineered blood vessels

**DOI:** 10.3389/fphys.2023.1210826

**Published:** 2023-05-12

**Authors:** Liang Wang, Zaozao Chen, Zhuoyue Xu, Yi Yang, Yan Wang, Jianfeng Zhu, Xiaoya Guo, Dalin Tang, Zhongze Gu

**Affiliations:** ^1^ State Key Laboratory of Bioelectronics, School of Biological Science and Medical Engineering, Southeast University, Nanjing, China; ^2^ Institute of Medical Devices (Suzhou), Southeast University, Suzhou, China; ^3^ School of Science, Nanjing University of Posts and Telecommunications, Nanjing, China; ^4^ Mathematical Sciences Department, Worcester Polytechnic Institute, Worcester, MA, United States

**Keywords:** tissue-engineered blood vessel, fluid–structure interaction, organ-on-a-chip, biomechanics, material properties

## Abstract

The tissue-engineered blood vessel (TEBV) has been developed and used in cardiovascular disease modeling, preclinical drug screening, and for replacement of native diseased arteries. Increasing attention has been paid to biomechanical cues in TEBV and other tissue-engineered organs to better recapitulate the functional properties of the native organs. Currently, computational fluid dynamics models were employed to reveal the hydrodynamics in TEBV-on-a-chip. However, the biomechanical wall stress/strain conditions in the TEBV wall have never been investigated. In this paper, a straight cylindrical TEBV was placed into a polydimethylsiloxane-made microfluidic device to construct the TEBV-on-a-chip. The chip was then perfused with cell culture media flow driven by a peristaltic pump. A three-dimensional fluid–structure interaction (FSI) model was generated to simulate the biomechanical conditions in TEBV and mimic both the dynamic TEBV movement and pulsatile fluid flow. The material stiffness of the TEBV wall was determined by uniaxial tensile testing, while the viscosity of cell culture media was measured using a rheometer. Comparison analysis between the perfusion experiment and FSI model results showed that the average relative error in diameter expansion of TEBV from both approaches was 10.0% in one period. For fluid flow, the average flow velocity over a period was 2.52 cm/s from the FSI model, 10.5% higher than the average velocity of the observed cell clusters (2.28 mm/s) in the experiment. These results demonstrated the facility to apply the FSI modeling approach in TEBV to obtain more comprehensive biomechanical results for investigating mechanical mechanisms of cardiovascular disease development.

## 1 Introduction

Organ-on-a-chip (OOC) is an artificial organ system that uses a microfluidic cell culture device to recapitulate the key biological and physiological functions of tissues and organs (Bhatia and Ingber, 2014; Zheng et al., 2016). During the past decade, different types of OOCs, including the blood vessels, lungs, heart, and blood–brain barrier, among others have been developed to model their *in vivo* counterparts in health and disease for replacement of animal testing and tissue-engineered drug development (Zheng et al., 2016; Yang et al., 2022). Blood vessel-on-a-chip is one of the extensively investigated OOCs, given its fundamental importance in cardiovascular pathophysiology (Yasotharan et al., 2015; Song et al., 2018; Su et al., 2021).

Great efforts have been exerted to develop various types of artificial blood vessel and blood vessel-on-a-chip, following the tissue-engineered approach (Niu et al., 2014; Niklason and Lawson, 2020). Early development of the tissue-engineered blood vessel (TEBV) based on synthetic polymer has been proved to be clinically useful to replace large internal diameter (>6 mm) arteries. In a clinical trial study, Hibino et al. have reported that tissue-engineered vascular grafts made by polyglycolic acid and e-caprolactone or L-lactide and seeded with autologous bone marrow-derived mesenchymal stem cells could be successfully implanted into patients as cardiac pulmonary conduits, and all grafts remained with the patient 1 year after implantation (Hibino et al., 2010). A small-diameter TEBV was also developed with collagen scaffold seeded with human umbilical cord blood-derived endothelial progenitor cells and umbilical artery smooth muscle cells (Chen et al., 2018). During the perfusion experiment, this TEBV exhibited elevated monocyte adhesion to the vessel wall when its endothelium was activated by the inflammatory cytokine. For disease modeling, a vascular microphysiological system using branched TEBV to emulate early atherosclerosis showed great promise to understand the pathological mechanisms to cardiovascular diseases (Lee et al., 2021). However, to faithfully recapitulate the blood vessels in health and disease, TEBV has to feature biomimetic structural, mechanical, chemical, and electrical environments as native ones (Zhang et al., 2017).

Currently, increasing attention has been paid to the biomechanical cues, like wall shear stress or cyclic strain/stress in tissue-engineered organs development and their impacts on the cell behaviors (Guilak et al., 2014). Engbers-Buijtenhuijs et al. (2006) investigated the SMC behaviors in both pulsatile and static flow conditions. They found that, compared to static conditions, TEBV cultured in dynamic conditions showed higher SMC numbers and these cells were more homogeneous distributed throughout the scaffolds. However, most of these biomechanical cues cannot be accurately measured in a quantitative way *via* remote sensing techniques (Pisapia et al., 2022). Thus, the computational modeling approach has been employed to simulate the biomechanical conditions inside the microfluidic channels for better tissue-engineered organs or OOC design (Patrachari et al., 2012). To optimize the shape of branched TEBV to implant into porcine models, Yeung et al. (2020) employed computational fluid dynamics (CFD) analysis to optimize the shape design of the custom-made TEBV for implantation with desired prognosis obtained. Hynes et al. (2020) examined circulating tumor cell behavior within a 3D-bioprinted vasculature to demonstrate that hydrodynamics simulated from a 3D computational flow model would determine the sites of vascular colonization. All aforementioned computational models do not consider the elasticity of the TEBV, which also greatly impacts the biomechanical environment (Guilak et al., 2014).

In this paper, a TEBV was fabricated to construct a blood vessel-on-a-chip. The elastic TEBV exhibited a periodic contraction and expansion movement in the circumferential direction when perfused with pulsatile flow of cell culture media *via* a peristaltic pump. Flow rate and pressure measurements were recorded in the perfusion experiment. The fluid–structure interaction (FSI) model was constructed to simulate the biomechanical conditions inside the TEBV, along with its cyclic movement and flow velocity. Comparison analysis between FSI simulation and experimental measurements were performed to validate the computational modeling approach. This work demonstrates a new approach combining OOC and FSI modeling to reveal the biomechanical characteristics in TEBV, which could be employed to investigate the biomechanical mechanisms of cardiovascular diseases and design optimization for TEBV-on-a-chip.

## 2 Materials and methods

### 2.1 Cell culture

Human umbilical vein endothelial cells (HUVECs) and human aortic smooth muscle cells (HASMCs) were obtained from the ScienCell Research Laboratories and cultured according to the established cell culturing protocols (Chen et al., 2018). HUVECs were cultured in endothelial cell medium (ScienCell), while HASMCs cultured in smooth muscle cell medium (ScienCell). When reaching 80% confluency, the cells were trypsinized (0.05% trypsin/EDTA; Thermo Fisher) and passaged. All cells were maintained in standard culture conditions (37 °C in humidified air with 5% CO_2_), and all cells were used within six to eight passages after reception to fabricate the TEBV.

### 2.2 Fabrication of the tissue-engineered blood vessel and blood vessel-on-a-chip

The vessel construct of TEBV was fabricated as a cylindrical shape similarly as previously described (Lee et al., 2021). Briefly, HASMCs were embedded in gelatin methacryloyl (GelMA) hydrogel with a concentration of 3.6 × 106 cells in 3 mL, and the cell and gel mixture was injected into a cylindrical mold with a mandrel in the middle for gelation (Ji et al., 2016; Chen et al., 2018). The outer and inner diameters of the TEBV were decided by the size of the mandrel and the mold, which were 5.0 mm and 2.0 mm, respectively. After gelation for 1 min by blue visible light, TEBV was placed into a polydimethylsiloxane (PDMS)-made microfluidic device to construct the blood vessel-on-a-chip. TEBV in the chip was supplied with smooth muscle cell media to mature for 1 week. After that, HUVECs were injected onto the inner surface of TEBV for coating. Finally, the TEBV was perfused with culture media for another 2 days to form functional endothelium.

### 2.3 Uniaxial tensile testing to characterize the mechanical properties of TEBV

To characterize the mechanical properties of TEBV under large deformation, uniaxial tensile testing was performed to obtain its stress–strain relationship (Teng et al., 2014). Four samples of freshly fabricated TEBV were trimmed into a dog-bone shape prior to the test. Sample thickness was measured by averaging the thickness values at four different locations. These samples were mounted on the tensile testing system (IPBF-300, CARE Measurement & Control) using fisher hook clamps (Lin et al., 2017). After preconditioning, tensile testing was carried out in force-control manner by stretching two clamps using a force from 0 to 0.02 N with a speed of 0.0002 N/s. The length (L) of the TEBV in the longitudinal direction and the applied tensile force (F) were both recorded to calculate the stress–strain (σ-λ) data

λ=L/L0, (1)

σ=F/D*h, (2)

where L is the time-varying length of the sample under different applied force F. L0 is the original length of the sample, D and h are the width and thickness of the sample, respectively.

### 2.4 Imaging of the peristaltic pump flow through TEBV-on-a-chip

A perfusion experiment was performed on TEBV-on-a-chip to investigate the TEBV movement when perfused with cell culture media flow. The vessel lumen of TEBV was connected to two perfusion ports *via* surgical suture as the inlet and outlet of the chip. In the experiment, a multi-channel peristaltic pump (BT100-1L, Longer Pump) was used to infuse the endothelial cell medium into the inlet and withdraw the fluid through the outlet simultaneously. Some cell clusters were added into the cell culture media to visualize the fluid flow. The segment of TEBV that was perfused with cell culture media was about 20.0 mm long, and it exhibited a cyclic circumferential movement under pulsatile flow driven by the peristaltic pump. The volumetric flow rate at the inlet was controlled by the peristaltic pump, and the pressure at the outlet was measured with a pressure sensor (LFT6800, Lefoo). The cyclic movement of TEBV was recorded by an Olympus IX83 inverted microscope.

### 2.5 Viscosity measurement of cell culture media

The viscosity of fresh cell culture media was measured using a rheometer (DHR-2, TA Instruments) at a shear rate range from 1.0–1000.0 s^−1^ (Poon, 2022). An adjunctive temperature control chamber was set at 37 °C. Data on shear stress (τ) corresponding to different shear rates (γ) were recorded for further analysis.

### 2.6 TEBV-based fluid–structure interaction model

To simulate the biomechanical conditions in TEBV, a three-dimensional (3D) FSI model was generated to fully mimic both the dynamic TEBV movement and pulsatile cell culture media flow (Yang et al., 2009). In the model, the fluid flow was assumed to be laminar and incompressible. The Navier–Stokes equations with arbitrary Lagrangian–Eulerian (ALE) formulation served as the governing equations. For the structural part, the TEBV was assumed to be an elastic homogeneous material. The governing equations of the structural model included the equation of motion, the non-linear Cauchy-Green strain-displacement relation, and stress–strain relationship. The inlet flow rate was prescribed at the inlet of the TEBV while measured pulsating pressure condition was prescribed at the outlet. No-slip boundary conditions and natural traction equilibrium conditions were imposed at the fluid–TEBV interface. By putting all mathematical equations together

$$\rho \left(\partial u_{i}/\partial t+\left(u_{j}-u_{gj}\right)u_{i,j}\;\right)=-p_{,i}+\mu \nabla^{2}u_{i}{,}_{jj},$$ (3)

$$u_{i,i}=0,$$ (4)

$$u\;|\Gamma =\partial x/\partial t,\partial u/\partial n{|}_{inlet,outlet}=0,$$ (5)

$$p{|}_{inlet}=p_{\mathrm{in}}\left(t\right),p{|}_{outlet}=p_{\mathrm{out}}\left(t\right),$$ (6)

$$\rho \;v_{i,tt}=\sigma_{ij,j},i,j=1,2,3;\;sum\;over\;j,$$ (7)

$$\varepsilon_{\mathrm{ij}}=\left(v_{i,j}+v_{j,i}+v_{\alpha ,i}\;v_{\alpha ,j}\right)/2,i,j,\alpha =1,2,3,$$ (8)

$$\sigma_{\mathrm{ij}}\cdot n_{j}{|}_{out\_wall}=0,$$ (9)

$$\sigma_{\mathrm{ij}}^{r}\cdot n_{j}{|}_{interface}=\sigma_{\mathrm{ij}}^{s}\cdot n_{j}{|}_{interface},$$ (10)

where **u** = (u1, u2, and u3) and p are the fluid velocity and pressure, **u**_g_ is the mesh velocity, µ is the dynamic viscosity, ρ is the density, Γ stands for the vessel inner boundary, **x** is the current position, **σ** is the stress tensor, **ε** is the strain tensor, **v** is the solid displacement vector, respectively, and superscript letters “r” and “s” were used to indicate different materials. The FSI models were solved using a commercial finite element package ADINA (ADINA R&D, Inc.) using the Newton–Raphson iteration method for mechanical conditions in both fluid and TEBV. Mesh analysis was performed by gradually refining the mesh density by 10% until changes of solutions became less than 2% (measured in L2-norm) (Yang et al., 2009). The final fluid mesh contains 224,886 tetrahedral elements, while the TEBV mesh has 36,540 elements in the hexahedral or prismatic shape. The time step was set as 0.01, following the previously established procedure to obtain stable numerical results (Wang et al., 2015).

## 3 Results

**FIGURE 1:**
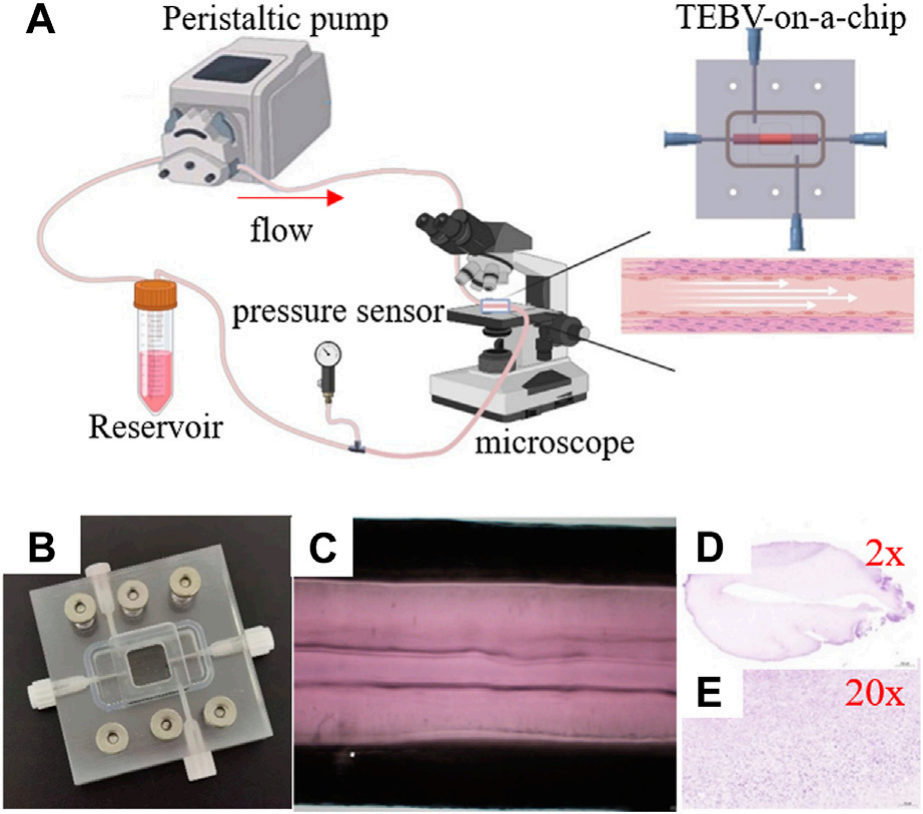
Imaging of the peristaltic pump flow through the tissue-engineered blood vessel. **(A)** Experiment setup. **(B)** TEBV-on-a-chip. **(C)** Image of TEBV under the perfusion experiment. **(D,E)** Images of TEBV with H&E staining.

Figure 1 shows the schematic illustration of the system to perform the TEBV perfusion experiment. More specifically, the inlet and outlet of blood vessel were connected to the cell culture media reservoir using plastic tubes to form a closed-loop of fluid flow. The FSI model was constructed to simulate the biomechanical conditions and was validated by comparing to the measurements of TEBV movement and flow velocity of cell culture media from the perfusion experiment. More details are given in the following subsections.

### 3.1 Construction of TEBV-on-a-Chip

**FIGURE 2:**
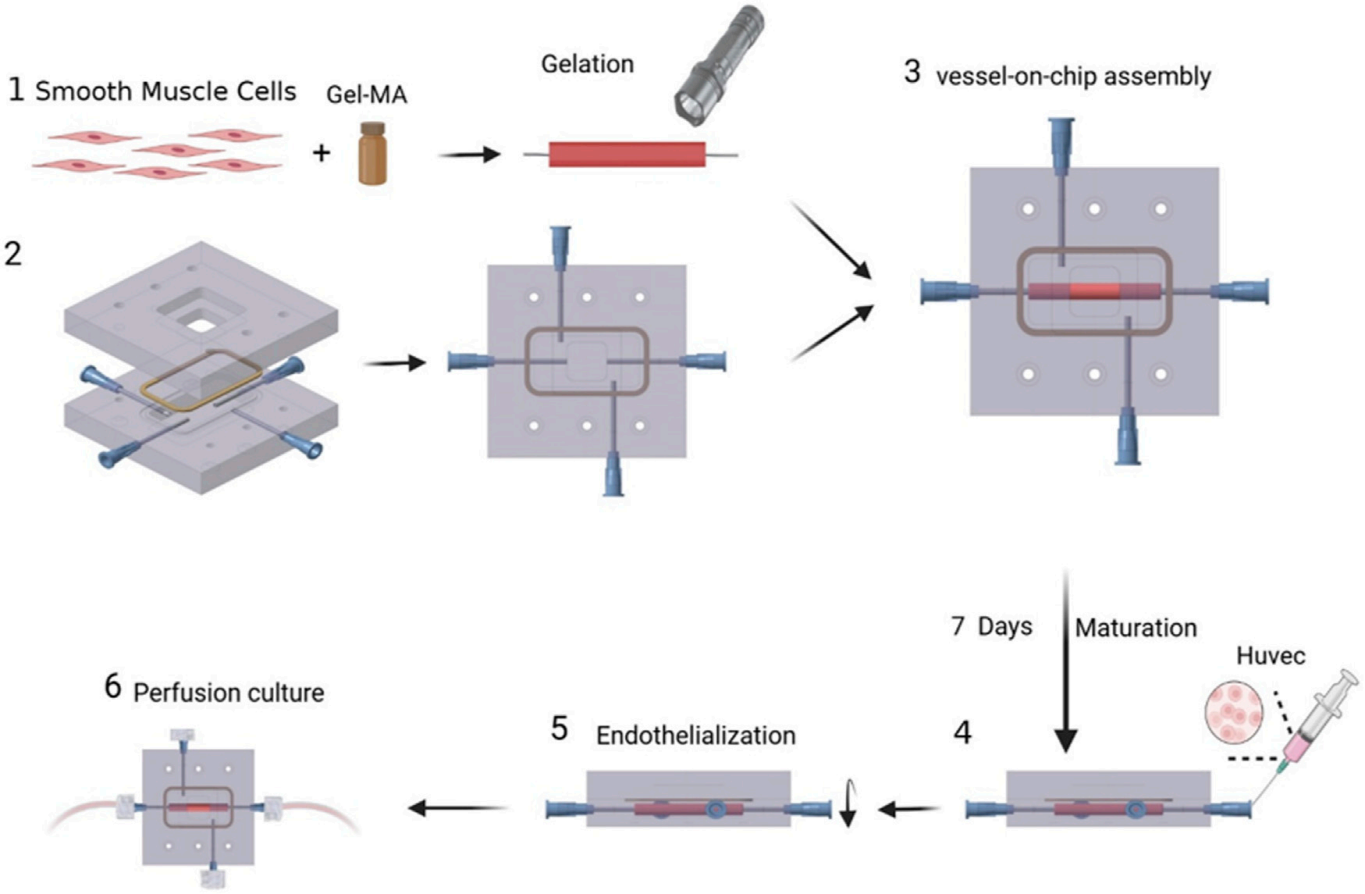
Schematic illustration of the construction of TEBV-on-a-chip.

The vessel construct of TEBV was fabricated in a straight cylindrical shape, with the inner and outer diameters to be 5.0 mm and 2.0 mm, respectively. It contains HASMCs in the vessel construct and seeded with HUVEC cells on the inner surface. Hematoxylin and eosin (H&E) staining was performed *via* frozen sections to confirm the presence of the cells (Figures 1D, E). A microfluidic device was fabricated by PDMS with a gel-molding chamber and a perfusion chamber. TEBV was placed inside the gel-molding chamber to construct the TEBV-on-a-chip with surgical suture (Figure 2). The vessel lumen of TEBV was connected to two perfusion ports as the inlet and outlet for the perfusion flow to go through (Figure 1A).

### 3.2 Quantification of TEBV movement and fluid flow

**FIGURE 3:**
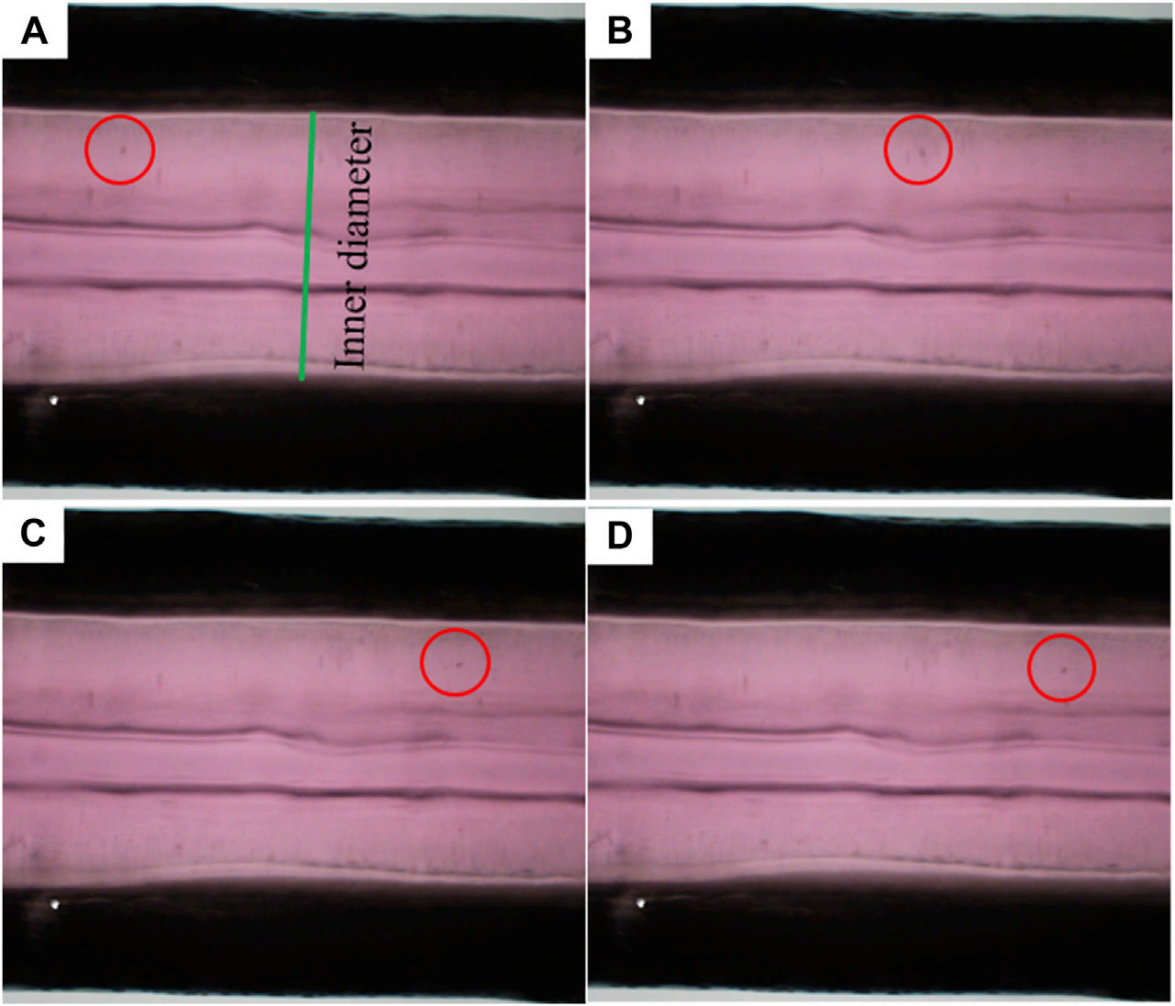
Movement of a sample flowing cell cluster at four different time instances. **(A–D)** Red circles indicate the locations of the cell cluster.

In the perfusion experiment, TEBV-on-a-chip was perfused with pulsatile flow of cell culture media driven by the peristaltic pump. The flow rate was set to 500 uL/min with 1 Hz pulse. The pressure sensor at the outlet of the TEBV showed that the measurement was varying from 640 to 780 Pa. Due to the pulsatile pressure condition, the TEBV exhibited a cyclic movement with circumferential contraction and expansion (Supplementary Video S1). The time-varying diameter of the TEBV at one cross-section was measured by calculating the distance between two TEBV-fluid interfaces on the images in one period (Figure 3). The flow velocity could be visualized by movement of the cell clusters, and the average velocity of the observed cell clusters (*n* = 5) was 2.28 (±1.80) mm/s in flow direction. Figure 3 shows the locations of the same cell cluster at four different instances.

### 3.3 Mechanical characterization of TEBV materials

**FIGURE 4:**
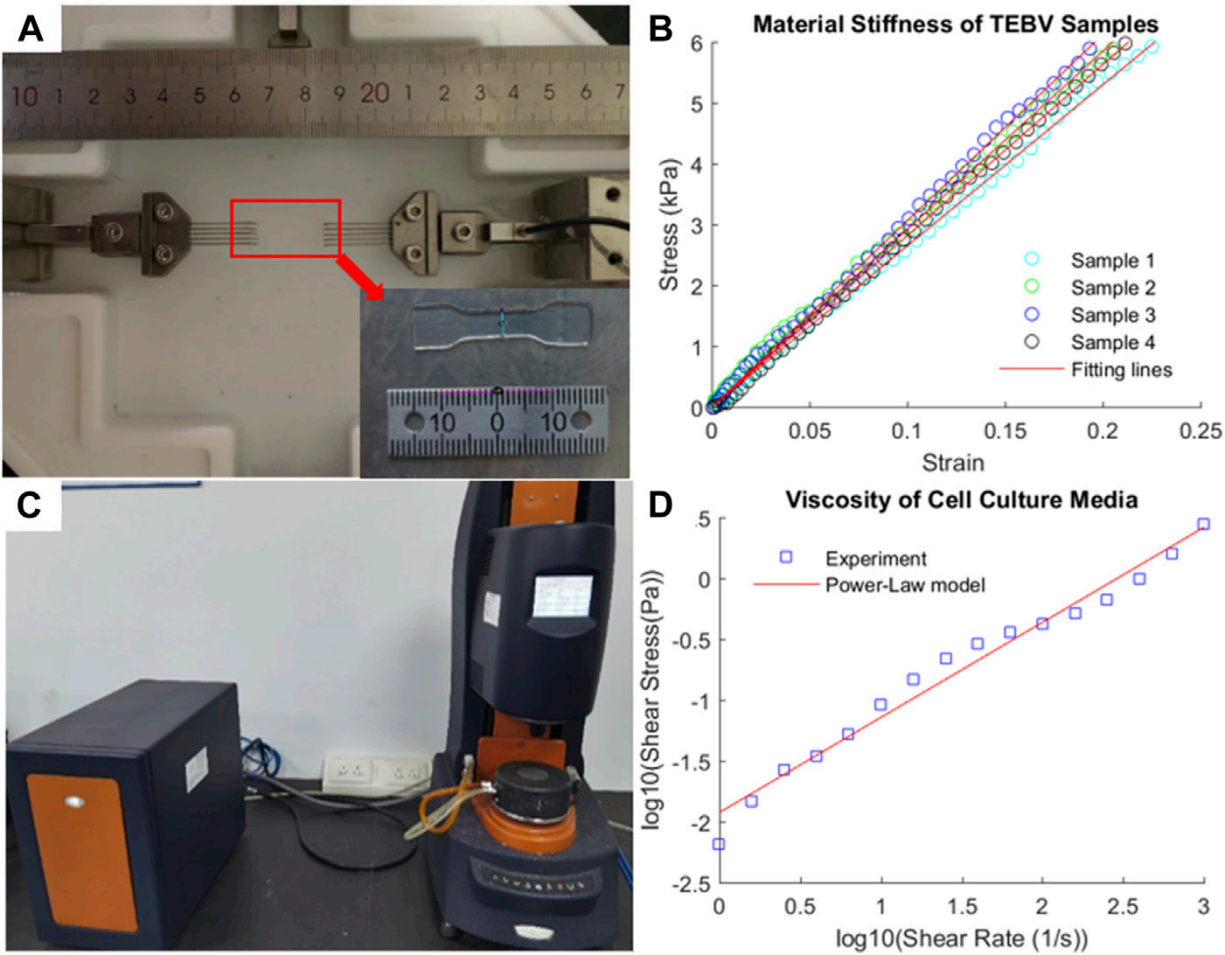
Experimental measurement of mechanical stiffness of TEBV and viscosity of cell culture media. **(A)** Uniaxial tensile testing of a TEBV sample in dog-bone shape; **(B)** stress–strain data from tensile testing and fitting line; **(C)** rheometer to measure the fluid viscosity; **(D)** shear stress-shear rate data and fitting line.

The experimental stress–strain data from four TEBV samples from uniaxial tensile testing are plotted in Figures 3A,B. Given the linearity of the stress–strain data, the linear Hookean material model (σ = E*λ) was chosen as the constitutive model to fit the stress–strain data (Karimi et al., 2013). The Young’s modulus (denoted as E) of the TEBV was estimated by fitting the data points in least square sense. The estimated Young’s modulus values for four TEBV samples are 26.5, 29.2, 30.6, and 28.1 kPa, respectively, with an average value of 28.6 (±1.73) kPa. The fitted linear curves for all four samples are also given in Figure 4B.

### 3.4 Cell culture media viscosity

The viscosity of the fluid was measured at a different shear rate ranging from 1 to 1000 s^−1^ (Figures 4C,D) to characterize the viscous properties of the cell culture media. Given the viscosity of the fluid is decreasing as the shear rate elevated, the fluid was assumed as a shear-thinning non-Newtonian fluid. The power-law model [log(τ) = log(K) + *n* log(γ)] was used to describe the relationship between shear stress and shear rate. After log transform of the shear stress and shear rate data, the least square method was employed to fit the data point to determine the material constants: K = 11.885 cp and *n* = −0.2188.

### 3.5 FSI simulation of the TEBV perfusion experiment

**FIGURE 5:**
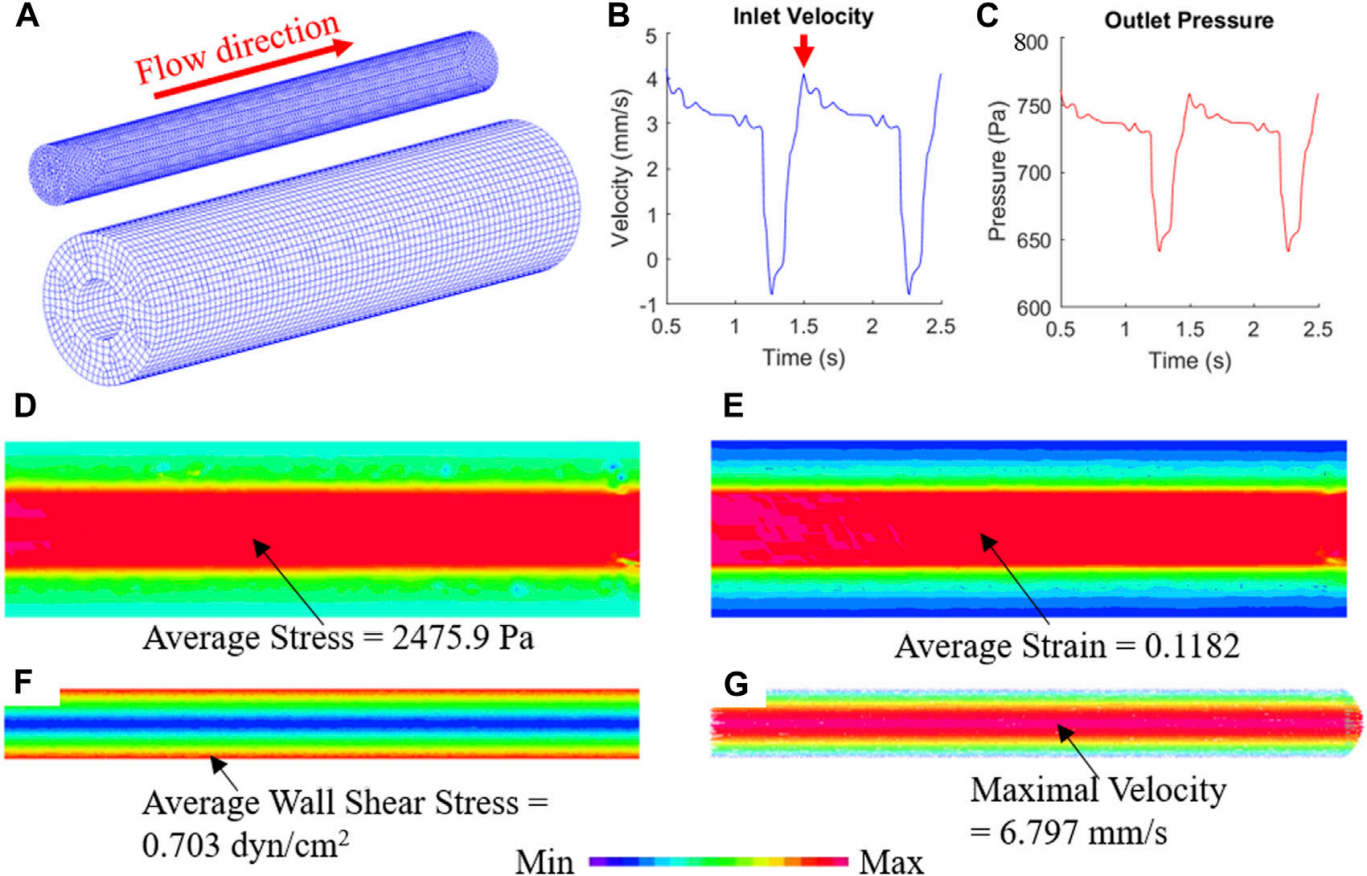
FSI simulation reveals the biomechanical conditions in TEBV and cell culture media. **(A)** Finite element mesh of TEBV and fluid model; **(B)** Inflow velocity profile at the inlet; **(C)** pressure profile at the outlet; **(D,E)** stress and strain conditions in the TEBV wall; **(F,G)** wall shear stress and velocity field in the cell culture media.

The biomechanical conditions in TEBV and cell culture media flow can be simulated by the FSI model (Figure 5A). To mimic the periodic pulsatile inflow driven by the peristaltic pump, the profile of inlet flow velocity follows a previous published waveform with a mean velocity of 2.65 mm/s to match the flow rate of 500 mL/min (Figure 5B) (Abello et al., 2022). The red arrow indicates the time point at which the flow velocity was at its maximum. At the outlet, the pressure condition with same sinuous function shape ranging from 640 to 780 Pa was prescribed (Figure 5C). After solving the computational model, detailed biomechanical conditions like fluid flow shear stress in the cell culture media and stress/strain distributions in the TEBV could be obtained, as well as the flow pattern in the fluid and cyclic movement of TEBV. Figures 5D–G shows the max principal stress/strain conditions in TEBV, and the velocity and flow shear stress in the fluid at a time point indicated by the red arrow. More details of biomechanical results over one whole cardiac cycle are given in Supplementary Video S2.

### 3.6 Comparison analysis between the perfusion experiment and FSI simulation

**FIGURE 6:**
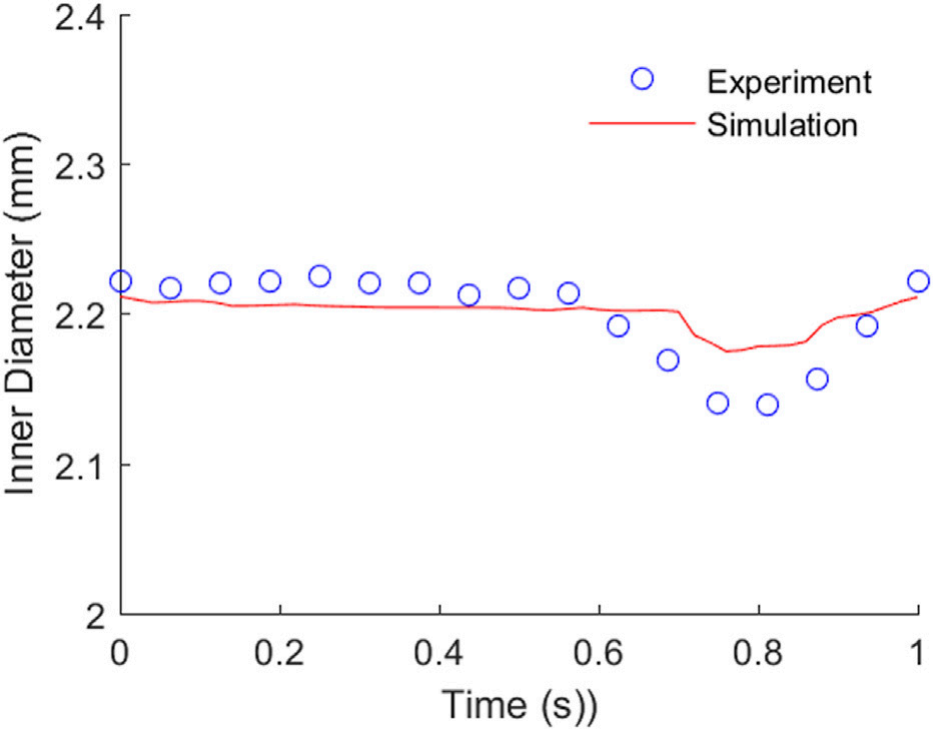
Comparison of the TEBV inner diameter movement from the perfusion experiment and FSI simulation.

The comparison of TEBV movement from the perfusion experiment and FSI simulation are plotted in Figure 6. The measured inner diameter of TEBV during the experiment suggested that its circumferential movement was cyclic. The change in vessel diameter during one period was larger than that from the FSI simulation, but a similar trend was observed in two approaches. The largest error in diameter difference occurs at around t = 0.8125 s. In the experiment, the inner diameter increased 0.14 mm from baseline 2.0 mm, while in the simulation, the diameter expanded 0.18 mm. The relative error is 28.6%. Over the whole period, the average value of this relative error is 10.0% between the experiment and the simulation. For the fluid flow, the flow rate from the FSI model is 474.78 uL/min, which is very close to the pre-set flow rate (500 uL/min) in the experiment (relation error is 4.95%). The average flow velocity over the cross section is 2.52 cm/s, which is 10.5% higher than the average velocity of the observed cell clusters (2.28 mm/s). The experimental value is slightly lower maybe due to the fact that the fluid velocity over the cross-section is non-uniformly distributed. If more cell clusters chosen were located near the TEBV wall, then the average velocity would be smaller. Nevertheless, the comparison analysis showed that both the TEBV movement and fluid dynamic from the experiment and FSI model were relatively close.

## 4 Discussion

### 4.1 Tissue-engineered blood vessel and other artificial blood vessels

Cardiovascular diseases remain to be the No. 1 killer all over the world, which involves the pathological diseases of the relevant arteries or veins. In clinical setting, some cardiovascular diseases are treated with an alternative substitute of the blood vessel to replace the diseased arteries, such as coronary artery bypass graft. Therefore, many artificial blood vessels were created and manufactured using synthetic polymer, rubber, hydrogel, etc., (Tara et al., 2014). Even though these substitutes resemble some properties to the native blood vessels, like mechanical stiffness or mechanical strength, they failed to replace the diseased blood vessel in clinical setting due to other drawbacks in biological and physiological functions (Camasão and Mantovani, 2021). TEBV is an emerging technology and shows great promise to fabricate the artificial blood vessels to possibly recapitulate the biological, biochemical, and biomechanical properties of the native ones.

TEBV fabricated here has several advantages over the traditional artificial ones in the following aspects: 1) Endothelialization of the TEBV to inhibit thrombosis; 2) seeded with HASMC cells to have relevant biological functions; and 3) TEBV possesses proper biomechanical strength and stress–strain relationship. Based on the uniaxial tensile testing experiment, it does exhibit elastic mechanical properties. In addition, it also has relevant strong biomechanical stiffness and material strength. Thus, TEBV-on-a-chip constructed here would be a more suitable alterative for cardiovascular diseases modeling and preclinical drug screening.

### 4.2 Fluid–structure interaction modeling of TEBV

The biomechanical stimulations are critical factors affecting the optimization of TEBV design or other tissue engineering organs. Unfortunately, many of these biomechanical cues cannot be measured using sensing techniques, and computational models provide a feasible mean to better reveal the exquisite biomechanical environment in the TEBV wall and fluid flow. Prior studies normally used CFD analysis to investigate the impact of microfluidic channel designs on hydrodynamic environment in OOCs (Pisapia et al., 2022). However, it cannot provide information on structural mechanics. Existing evidence has shown that these structural mechanics are also vital for optimizing the biological functions of OOCs, especially in lung-on-a-chip and heart-on-a-chip (Huh et al., 2010; Marsano et al., 2016). These mechanical stimulations could enhance cellular behaviors and the cell–cell or cell–scaffold interaction to better mimic the physiological function of the organs. To the best of our knowledge, this is the first FSI modeling study to obtain the mechanical conditions in tissue-engineered organs in a quantitative way. This modeling approach based on tissue-engineered organs would serve as a powerful tool to investigate the biomechanics and study its impact on functional properties of tissue-engineered organs.

### 4.3 TEBV-based biomechanical modeling for cardiovascular disease investigation

It is well known that biomechanical factors pose strong influence on biological behaviors of the cells in the organs and thus may lead to tissue remodeling or even pathological development (Haga et al., 2007). For instance, cardiovascular diseases like stroke or heart attack are caused by the atherosclerosis of the carotid and coronary arteries. The initiation, progression, and rupture of atherosclerosis are affected by the biomechanical conditions. Accumulating information has supported this conclusion in silicon and *in vivo* (Kwak et al., 2014). Currently, most published studies are based on *in vivo* clinical images from patients to simulate the biomechanical conditions and then investigate whether any correlation between the plaque behavior and biomechanics. However, one limitation regarding to *in vivo* clinical studies is that we could neither recruit enough typical clinical cases to have a full picture of the process of plaque rupture *in vivo* nor could we have a good animal model to recapitulate the human arterial atherosclerosis. TEBV is a good substitute for such case to replace the animal model to exhibit the pathological development of atherosclerosis. Branched TEBV with early atherosclerosis has also been developed (Lee et al., 2021). Furthermore, the TEBV-based perfusion experiment could provide a controllable biomechanical environment to study their impacts on disease development. Varying biomechanical conditions could be easily achieved by adjusting the flow rate, TEBV stiffness, or changing pressure conditions at the outlet. Thus, combining *in vitro* experiments on tissue-engineered organs and computational models would provide a better approach to understand the impact of biomechanics on the process of initiation, progression, and rupture of atherosclerotic plaque, and could also be extended to study other cardiovascular diseases.

## 5 Conclusion

In this work, we first applied FSI modeling on TEBV to simulate the comprehensive biomechanical conditions, including stress/strain conditions in TEBV and wall shear stress in cell culture media flow during the perfusion experiment. Comparison analysis between FSI simulation results and the perfusion experiment was performed to warrant the facility of applying the FSI modeling approach in TEBV-on-a-chip to further study these biomechanical cues in cardiovascular diseases modeling and OCC designs.

## Data Availability

The original contributions presented in the study are included in the article/Supplementary Material; further inquiries can be directed to the corresponding authors.
